# Association between patient care ownership and personal or environmental factors among medical trainees: a multicenter cross-sectional study

**DOI:** 10.1186/s12909-022-03730-y

**Published:** 2022-09-09

**Authors:** Hirohisa Fujikawa, Daisuke Son, Takuya Aoki, Masato Eto

**Affiliations:** 1 Department of Medical Education Studies, International Research Center for Medical Education, Graduate School of Medicine, The University of Tokyo, 7-3-1 Hongo, Bunkyo-ku, Tokyo, 113-0033 Japan; 2 Department of Community-Based Family Medicine, Faculty of Medicine, Tottori University, Yonago, Tottori, Japan; 3 Division of Clinical Epidemiology, The Jikei University School of Medicine, Minato-ku, Tokyo, Japan; 4 Section of Clinical Epidemiology, Department of Community Medicine, Graduate School of Medicine, Kyoto University, Sakyo-ku, Kyoto, Japan

**Keywords:** Patient care ownership, Patient ownership, Personal factors, Environmental factors, Working hour, Duty hour restriction, Duty hour regulation, Workplace, Learning environment

## Abstract

**Background:**

Patient care ownership (PCO) is crucial to enhancing accountability, clinical skills, and medical care quality among medical trainees. Despite its relevance, there is limited information on the association of personal or environmental factors with PCO, and thus, authors aimed to explore this association.

**Methods:**

In 2021, the authors conducted a multicentered cross-sectional study in 25 hospitals across Japan. PCO was assessed by using the Japanese version of the PCO Scale (J-PCOS). To examine the association between personal (level of training, gender, and department) or environmental factors (hospital size, hospital type, medical care system, number of team members, number of patients receiving care, mean working hours per week, number of off-hour calls per month, and perceived level of the workplace as a learning environment) and PCO after adjusting for clustering within hospitals, the authors employed a linear mixed-effects model.

**Results:**

The analysis included 401 trainees. After adjusting for clustering within hospitals, it was confirmed that the senior residents had significantly better J-PCOS total scores (adjusted mean difference: 8.64, 95% confidence interval [CI]: 6.18–11.09) than the junior residents and the perceived level of the workplace as a learning environment had a positive association with J-PCOS total scores (adjusted mean difference per point on a global rating of 0–10 points: 1.39, 95% CI: 0.88–1.90). Trainees who received calls after duty hours had significantly higher J-PCOS total scores than those who did not (adjusted mean difference: 2.51, 95% CI: 0.17–4.85). There was no clear trend in the association between working hours and PCO.

**Conclusions:**

Seniority and the perceived level of the workplace as a learning environment are associated with PCO. An approach that establishes a supportive learning environment and offers trainees a reasonable amount of autonomy may be beneficial in fostering PCO among trainees. The study findings will serve as a useful reference for designing an effective postgraduate clinical training program for PCO development.

## Background

Patient care ownership (PCO) is an important component of medical professionalism [1, 2]. The definition of PCO is an emotional and cognitive state in which physicians use emotional and intellectual factors to make decisions [1]. For trainees, increased ownership is expected to lead to increased accountability and improved clinical skills, which will improve the quality of patient care [1]. As such, PCO is widely recognized as an essential component of the professionalism that must be developed during medical training [3, 4].

Conventionally, PCO is described as “the philosophy that one knows everything about one’s patients and does everything for them [5].” However, in recent years, with international regulations on working hours of physicians, the concept of PCO has gradually been changed. For example, Cowley et al. identified the following topics as central to PCO in their qualitative research: advocacy; decision-making regarding treatment planning; communication and care coordination; monitoring; opinion-acquisition of patient awareness; leadership; doing more than the bare minimum requirement; thinking of oneself as the one who is ultimately responsible; acting as a provider of primary care; initiative taking; and providing the best care [6]. A qualitative study performed by Masson et al. identified three factors as key features of PCO in an internal medicine night float system: detailed knowledge of patients, autonomous decision-making, and continuous personal concern for patients [7]. Thus, PCO is now considered as a multifaceted concept.

The various factors that can affect PCO have been described in previous literature. Among them, the impact of working hours on PCO has often been discussed, but no firm conclusions have been reached thus far. Due to the introduction of global working hours restrictions for residents, many authors believe that system-based work styles may be accompanied by the acquisition of a “shift mentality” (a perception that the clock dictates the medical resident’s departure time from hospital, not the patient’s needs), which adversely affects the development of professional identity and PCO [5, 8, 9]. On the contrary, Szymczak et al., in their observational ethnographic study, indicated that a strong fear of working hours restrictions and a decline in resident expertise due to the development of a “shift work” mentality are likely to be exaggerated. Moreover, the impact of working hours’ regulation on professionalism and PCO is more complex than public opinion [10]. Environmental factors other than working hours (e.g., faculty supervision, subspecialist involvement, and learning environment) and trainees’ individual factors (e.g., seniority, specialty, and work ethic) are also expected to influence PCO [11, 12]. However, these early studies were limited by the lack of validated quantitative tools for measuring PCO.

Recently, Djulbegovic et al. developed the PCO Scale (PCOS), which is a device for quantitatively measuring PCO in an inpatient setting [13]. This English version of PCOS has shown good reliability and validity in the United States [13, 14]. We have developed a Japanese version of PCOS (J-PCOS), that is, a translated and culturally adapted version of the original PCOS. This J-PCOS has been well validated among trainees from various clinical departments at several institutions across Japan [15]. These reliable and objective tools would be useful to quantitatively examine ownership.

Therefore, we aimed to quantitatively investigate the association of personal or environmental factors with PCO. The results of this study may provide the basis for developing effective educational interventions to promote ownership, possibly leading to enhancing the quality of patient care.

## Methods

### Context

In Japan, those who wish to practice clinical medicine enter obligatory clinical graduate program after obtaining a National License for Physicians. All trainees alternate between several clinical departments (junior residents; *kenshui* in Japanese) for two years. Only after the two years of training, doctors proceed to advanced graduate clinical training in specialized fields, which usually lasts more than three years (senior residents; *senkoui* in Japanese) [16].

### Study design

In this study, we used a multicenter cross-sectional design. Approval for conducting this study was granted from the Institutional Review Board of the University of Tokyo (IRB approval number: 2021108NI).

### Setting and participants

Thirty graduate clinical training hospitals in Japan were selected using information from the Residency Electronic Information System, which is a database of teaching hospitals developed and maintained by the Ministry of Health, Labor, and Welfare of Japan. Twenty-five out of the thirty hospitals have agreed to collaborate with our research. Participating hospitals were geographically distributed throughout Japan. These hospitals were of different sizes and included both university and community hospitals (Table 1).

**Table 1:** Characteristics of the 25 participating hospitals

Characteristics	N (%)
Hospital sizes	
≤ 500 beds	15 (60)
501–800 beds	5 (20)
801–1000 beds	2 (8)
≥ 1001 beds	3 (12)
Hospital types	
Community hospital	20 (80)
University hospital	5 (20)
Hospital locations	
Hokkaido and Tohoku	4 (16)
Kanto	3 (12)
Chubu	3 (12)
Kinki	4 (16)
Chugoku	3 (12)
Shikoku	3 (12)
Kyushu	5 (20)

Anonymous questionnaires were distributed to all the eligible participants [*n* = 1038] by their training program administrators in September 2021. The eligible participants in the study were all trainees in their first through sixth post-graduate years (PGYs) in the training programs of the 25 hospitals that had committed to participate in the study. The participants completed the questionnaires, put them in their respective envelopes and handed them to the training program administrators. The administrators mailed them to the researchers. The participants were informed that participation was voluntary. About a week after the survey was distributed, they received a reminder. A second reminder was sent a week later.

### Outcome variable: Patient Care Ownership Scale

Patient Care Ownership was measured using J-PCOS [15]. J-PCOS is a 13-item instrument. Participants were asked to assume inpatient care settings when completing the survey. Each item was rated on a 7-point Likert scale ranging from 1 = strongly disagree to 7 = strongly agree. The total scores of J-PCOS were calculated by totaling the scores of the item for each participant. Therefore, the total J-PCOS ranges from 13 to 91, with higher values indicating better PCO.

### Environmental factors

Based on a literature review [7, 11–14, 17], various environmental factors that might be associated with PCO were included as explanatory variables: hospital size (≤ 500 beds; 501–800 beds; 801–1000 beds; or ≥ 1001 beds), hospital type (community hospital vs. university hospital), medical care system (single *shujii* system vs. multiple *shujii* system (as will hereinafter be described in detail)), number of team members (≤ 2; 3–4; 5–6; or ≥ 7), number of inpatients in charge (≤ 3; 4–6; 7–9; or ≥ 10), average working hours per week, number of calls during off-hours per month (0 vs. ≥ 1), and the perceived level of the workplace as a learning environment.

With regards to the question of medical care system, participants were asked to choose between two of the following options: a single *shujii* (the doctor primarily responsible for the patient) system and multiple *shujii* system (team system). In Japan, most hospitals used a single *shujii* system. In a single *shujii* system, a single physician takes charge of a patient’s care until discharge. In this system, even if other physicians are on duty during their off-hours, hospital physicians cannot simply take a break because they must constantly check the patients’ conditions and treat them accordingly [18]. When a resident becomes a *shujii* for a given patient, the legal responsibility for patient care is considered to rest with the supervisor, but the majority of the management (e.g., examination planning, treatment planning, and explanation of medical condition to the patient) is left to the resident. However, in recent years, physicians being overworked has become a problem in Japan [19, 20], and some hospitals have begun to adopt multiple *shujii* system as a counter response. In multiple *shujii* system, multiple physicians are responsible for patient care as a team. As either of these two systems is likely to affect the trainees’ PCO development, we decided to include it as an explanatory variable in this study.

We inquired participants about the average working hours on weekends and weekdays and the number of night shifts per month they undertake. Based on previous studies [21–23], we calculated the average working hours per week using the following formula:

Average working hours per week = 5 * (Average working hours on weekdays) + 2 * (Average working hours on weekends) + 7 * (Number of night duties per month/30) * (24–Average working hours on weekdays).

Prior research (both qualitative and quantitative) has shown that the learning environment may have an impact on PCO training [12, 14]. In particular, the paper validating the PCO Scale showed that residents trained in a positive learning environment had a significantly higher PCO through a bivariate analysis using the Mini-Rez scale by Linzer et al. [14]. Accordingly, we decided to include the level of the workplace as a learning environment as an explanatory variable. We chose a single-item global rating scale for the following three reasons. First, in the field of research on medical education and working environment, the usefulness of global rating scales has been proposed due to their excellence in capturing nuanced elements [24–26]. The learning environment is a multifaceted concept, and its nuances may be better captured by a global rating scale. In fact, a one-item measure of the learning environment has been used in previous studies in the medical education field [27–29], which would justify our use of a global rating in this study. Second, in terms of response rates, shorter questionnaires generally yield better results [30]. Because the survey dealt with numerous explanatory variables, there was concern that the response rate would decline if a large number of questions were required for each explanatory variable. Third, to the best of our knowledge, there is no valid Japanese version of the Mini-Rez scale. Therefore, we used a single measure of the level of the workplace as a learning environment as follows: “Using any number from 0 to 10, where 0 is the worst learning environment possible and 10 is the best learning environment possible, what number would you use to rate your current department as a learning environment?”.

### Personal factors

We also included some personal factors as possible explanatory variables related to PCO based on previous studies [7, 11–14, 17]; they are as follows: level of training (PGY 1–2 (junior residents; *kenshui*) vs. PGY 3–6 (senior residents; *senkoui*)); participants’ gender (female; male; or other identities); and participants’ department (internal medicine; surgery; or other departments).

In Japan, internal medicine and surgery as well as other departments handle a large number of inpatients. We considered that differences by department might affect PCO and decided to add participants’ departments to the explanatory variables.

### Statistical analysis

In this study a linear mixed-effects model was employed (random intercept model), which includes random effects for hospitals and explanatory variables (i.e., gender, level of training, department, hospital size, hospital type, medical care system, number of team members, number of inpatients in charge, average weekly working hours, post-work on call obligations, and workplace level as a learning environment) as fixed effects. The complete case analysis approach was chosen because of the small amount of missing data. A two-tailed *p* value < 0.05 was considered to be statistically significant. We used SPSS Statistics 27.0 (IBM Japan; Tokyo, Japan) to analyze our data.

## Results

**Fig. 1:**
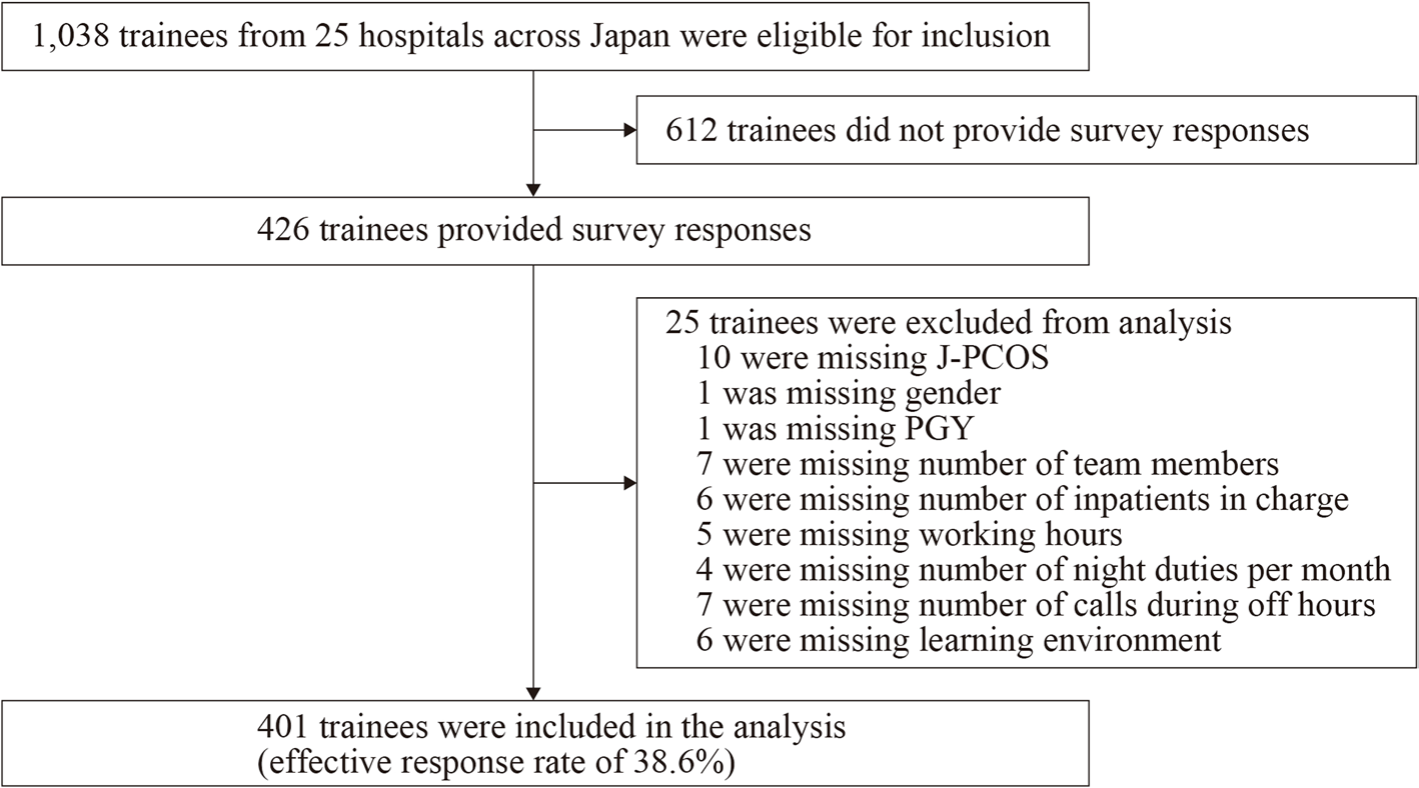
Flowchart of participants in a study of the association between patient care ownership and personal or environmental factors in trainees in Japan. J-PCOS, the Japanese version of Patient Care Ownership Scale; PGY, postgraduate years

Of the 1038 eligible trainees, 426 (41.0%) completed the survey. Due to lack of data, we excluded the responses from the 25 trainees. Therefore, 401 (38.6%) respondents were included in the analysis (Fig. 1). The average total J-PCOS score was 60.7 (SD = 11.7). Table 2 shows descriptive statistics for all the explanatory variables included.

**Table 2:** Characteristics of participants (*N* = 401)

Variable, unit of measure	Value
Gender, N (%)	
Female	132 (32.9)
Male	264 (65.8)
Others	5 (1.2)
Postgraduate Years (PGY), N (%)	
PGY 1–2	192 (47.9)
PGY 3–6	209 (52.1)
Department, N (%)	
Internal medicine	142 (35.4)
Surgery	43 (10.7)
Other departments	216 (53.9)
Hospital size, N (%)	
≤ 500 beds	185 (46.1)
501–800 beds	57 (14.2)
801–1000 beds	47 (11.7)
≥ 1001 beds	112 (27.9)
Hospital type, N (%)	
Community hospital	272 (67.8)
University hospital	129 (32.2)
Medical care system, N (%)	
Multiple *shujii* system (team system)	202 (50.4)
Single *shujii* system	199 (49.6)
Number of team members, N (%)	
≤ 2	119 (29.7)
3–4	132 (32.9)
5–6	79 (19.7)
≥ 7	71 (17.7)
Number of inpatients in charge, N (%)	
≤ 3	147 (36.7)
4–6	127 (31.7)
7–9	52 (13.0)
≥ 10	75 (18.7)
Average working hours per week, measn (SD)	69.21 (12.72)
Number of calls during off-hours per month, N (%)	
0	219 (54.6)
≥ 1	182 (45.4)
Perceived level of the workplace as a learning environment,^a^ mean (SD)	7.66 (1.94)

^a^Ranges from 0 to 10, with higher scores indicating a better learning environment

Table 3 shows the results of analysis of the association between personal or environmental factors and PCO using a linear mixed-effects model. In terms of personal factors, multilevel analysis revealed that the senior residents had significantly higher J-PCOS total scores (adjusted mean difference: 8.64, 95% confidence interval [CI]: 6.18–11.09) than the junior residents. Among the environmental factors, the level of perception of the workplace as a learning environment was significantly associated with J-PCOS total scores (adjusted mean difference per point on a global rating of 0–10 points: 1.39, 95% CI: 0.88–1.90). Trainees who received calls during off-hours had substantially higher J-PCOS total scores than those who did not (adjusted mean difference: 2.51, 95% CI: 0.17–4.85). There was no clear trend in the association between working hours and PCO.

**Table 3:** Multilevel linear regression of personal or environmental factors associated with J-PCOS

	**Adjusted mean difference (95% CI)**
Individual variables	
Gender (Ref: Male)	
Female	1.07 (-1.03 to 3.17)
Others	-3.54 (-12.48 to 5.39)
Level of training (Ref: PGY 1–2)	
PGY 3–6	8.64** (6.18 to 11.09)
Department (Ref: Surgery)	
Internal medicine	1.00 (-2.49 to 4.50)
Other departments	1.47 (-2.03 to 4.96)
Unit variables	
Hospital size (Ref: ≥ 1001 beds)	
≤ 500 beds	-1.24 (-5.67 to 3.19)
501–800 beds	-2.40 (-7.47 to 2.67)
801–1000 beds	-3.42 (-9.87 to 3.03)
Hospital type (Ref: University hospital)	
Community hospital	-1.03 (-5.67 to 3.62)
Medical care system (Ref: Multiple *shujii* system (team system))	
Single *shujii* system	1.46 (-0.86 to 3.79)
Number of team members (Ref: ≥ 7)	
≤ 2	0.11 (-3.24 to 3.45)
3–4	-1.00 (-4.06 to 2.05)
5–6	-0.76 (-4.12 to 2.60)
Number of inpatients in charge (Ref: ≤ 3)	
4–6	-0.23 (-2.66 to 2.21)
7–9	1.78 (-1.52 to 5.09)
≥ 10	1.76 (-1.24 to 4.77)
Average working hours per week	0.02 (-0.06 to 0.11)
Number of calls during off-hours per month (Ref: 0)	
≥ 1	2.51* (0.17 to 4.85)
Perceived level of the workplace as a learning environment	1.39** (0.88 to 1.90)

*Abbreviations*: *CI* Confidence interval, *J-PCOS* The Japanese version of Patient Care Ownership Scale, *PGY* Postgraduate years

Note: J-PCOS scores range from 13 to 91, with higher scores indicating better PCO. Perceived level of the workplace as a learning environment ranges from 0 to 10, with higher scores indicating a better learning environment. *p < 0.05, **p < 0.01

## Discussion

In this study, we found that the level of training and perceived level of the workplace as a learning environment are associated to PCO; whereas, there were no clear trends between working hours of doctors and PCO. The results of this study will be a useful reference for postgraduate medical education on PCO.

In this study, senior residents had significantly higher PCO than juniors. There are two possible reasons for this; first, senior residents can improve their PCO by gaining knowledge, skills, and self-confidence through years of clinical training. Second, when they become senior residents, trainees have more opportunities to play a clinical role. In recent years, concerns about patient safety have prompted calls for increased clinical supervision of trainees, especially those who are novice [31]. Strengthening supervision can undermine resident autonomy and hinder the development of a sense of ownership [32]. This is especially so in the Japanese postgraduate training system, because PGY 1–2 trainees need to be rotated through multiple departments and are assigned to one department for a short period of time. It can be difficult for supervisors to delegate clinical tasks to junior residents, which can limit autonomy. For balancing clinical supervision and resident autonomy, an existing model (the SUPERB/SAFETY model) may be useful [33, 34].

The finding that there is a significant positive association between the degree of workplace as a learning environment and PCO, supports the results of a previous study. The previous study has indicated that internal medicine residents who worked in a positive learning environment exhibited more ownership than those who did not [14]. The learning environment refers to the condition or surroundings in which learning takes place. The learning environment in which medical education takes place can have a significant impact on the learners; it is most often described as an integral part of the hidden curriculum. Hidden curriculum is defined as a series of impacts on the learning environment at the organizational structure and cultural level [35], and has been studied as a powerful force for professional development of trainees [36–39]. Therefore, PCO, an important element of professionalism, is considered to be strongly influenced by hidden curriculum and the learning environment. Past qualitative studies have suggested the importance of role models in the learning environment for the development of PCO among residents [7], but more details on which elements of the learning environment are involved in the development of PCO are not understood and need to be examined in the future.

The fact that there was no clear trend in the relationship between working hours and PCO is contrary to the expectations of many previous authors. Many have suggested that working hours restrictions can lead to shift-worker mentality and loss of professional identity and PCO [5, 8, 9]. Recently, it has been shown that regulations of working hours can bring about many positive changes. For example, in a qualitative descriptive study by proponents of internal medicine residency program compared to the previous 24-h call system [7], the night float system, as an exemplary application of working hour regulations, has less mental and physical fatigue, more consistent interaction with patients, and a more stable team structure within shifts. These changes in the work environment have been found to improve teamwork, quality of work, empathy, and ownership of patient care [7, 40, 41]. However, the association between long working hours and the educational outcomes of residents, including PCO, has rarely been fully investigated in Japan or elsewhere in the world [42]; hence, a more solid research is required.

This study aims to quantitatively explore the association between PCO and personal or environmental factors using the validated instrument. One of the strengths of this study is its robust methodology, that is, multilevel analysis of data from trainees nationwide. If individuals are nested within institutions, there are correlations within an institution that should be considered when analyzing data from individuals belonging to those institutions. Multilevel analysis allows researchers to better explain correlation within an institution. Traditional regression and analysis of variance methods are inadequate for dealing with intra-institutional correlations; therefore, they are generally not recommended in such situations [43]. An article examining the original English version of the PCOS showed that PCO was higher among senior residents and residents in a positive learning environment [14]. However, this study was limited in that it included only residents of the five residency programs, and performed a bivariate analysis of the difference in mean ownership due to the characteristics of the residents. In this study, we collected data from trainees from many hospitals across Japan. Because ownership of patient care varies from hospital to hospital, we used a linear mixed-effects model to coordinate clustering within the hospital to enable appropriate analysis at the individual level. Our work using this robust methodology reinforces the findings of the previous studies and has a relatively high external validity.

### Implications

This study indicated that the workplace as a learning environment rather than working hours was important for the development of PCO. In addition, the higher the resident’s PGY, the higher the PCO scores, which suggests the importance of providing a learning environment in which trainees can work independently. Strategies that create a supportive learning environment and provide trainees with a reasonable level of autonomy can be effective in fostering PCO. In the future, educational and programmatic structures must be developed with these strategies and verify the effectiveness of such programs.

### Limitations

This study has some potential limitations. First, although the results suggested an association between PCO and personal or environmental factors, a cross-sectional exploratory study design did not allow us to determine whether these associations were causal or not. Additional longitudinal hypothesis-testing studies are required to confirm causality. Second, working hours were self-reported in the study. One previous study found that self-reporting by residents was relatively accurate [44]; however, future studies require the use of automated attendance management system to measure working hours accurately. Third, the reliability and validity of the scale for the learning environment were unknown. Fourth, the response rate to the questionnaire was relatively low, which could lead to selection bias. Finally, participating hospitals voluntarily took part in the study, that is, the preset sample could be representative of hospitals with a higher interest in PCO. Thus, caution is required when generalizing the results of this study to other institutions.

## Conclusions

In this study, using a validated instrument, we investigated the association between PCO and personal or environmental factors among trainees across Japan. There was a positive association between PCO and seniority and between PCO and the level of the workplace as a learning environment, but no clear trend was identified between PCO and working hours. Since the participating hospitals were widely distributed throughout Japan, varied in size, and included both community and university hospitals, the results have relatively high external validity. For the development of trainees’ PCO, strategies that create a supportive learning environment and give them a reasonable amount of autonomy may be important. The findings will serve as a reference for developing an effective postgraduate clinical training program for nurturing PCO, which can lead to increased accountability among residents, improved clinical skills, and better quality of patient care.

## Data Availability

The datasets generated and/or analyzed during the current study are available from the corresponding author on reasonable request.

## Abbreviations

PCO: Patient care ownership
PCOS: Patient Care Ownership Scale
J-PCOS: Japanese version of the Patient Care Ownership Scale
PGY: Postgraduate years
CI: Confidence interval
